# Electrospray Deposition of Catalyst Layers with Ultralow Pt Loading for Cost-Effective H_2_ Production by SO_2_ Electrolysis

**DOI:** 10.1021/acsaem.1c03672

**Published:** 2022-02-04

**Authors:** Imen Fouzai, Maher Radaoui, Sergio Díaz-Abad, Manuel Andrés Rodrigo, Justo Lobato

**Affiliations:** † Laboratory of Technology, Energy, Materials and Innovation “TEMI”, Faculty of Sciences of Gafsa, Cité Sidi Ahmed Zarroug, 2112 Gafsa, Tunisia; ‡ National Institute of Applied Sciences and Technology, B.P. No. 676, 1080 Tunis Cedex, Tunisia; § Department of Chemical Engineering, Faculty of Chemical Sciences & Technologies, University of Castilla-La Mancha, Campus Universitario no. 12, 13071 Ciudad Real, Spain

**Keywords:** green H_2_ production, Westinghouse cycle, low Pt loading, electrospray deposition

## Abstract

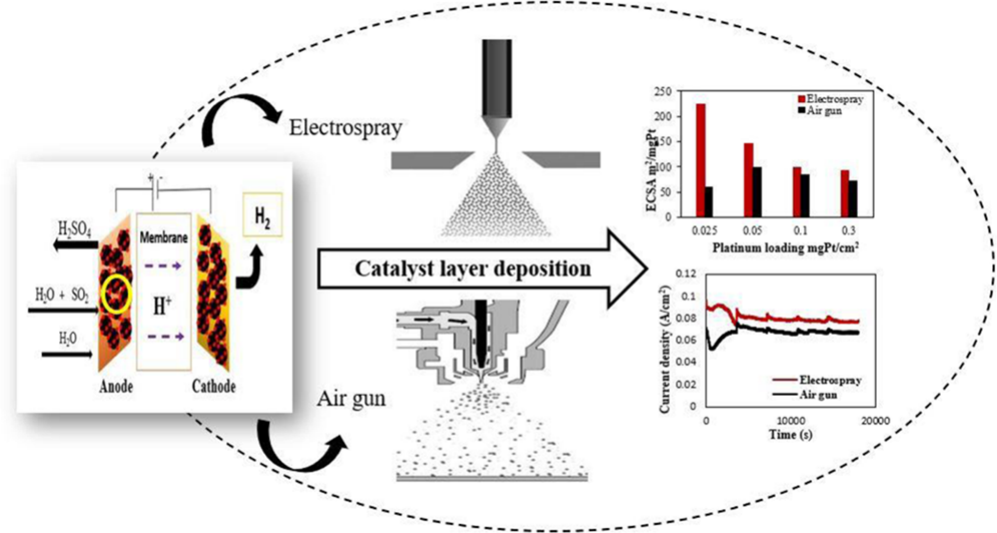

The hybrid sulfur (HyS) thermochemical cycle has been considered as a promising approach for the massive production of clean hydrogen without CO_2_ emissions. The key to advance this technology and to enhance the cycle efficiency is to improve the electrocatalytic oxidation of SO_2_, which is the pivotal reaction within this process. Hence, this paper investigates, for the first time, the effect of electrospray and air gun deposition techniques and the influence of very low Pt loadings (<0.3 mg Pt/cm^2^) on catalyst durability and activity. The variation of electrochemical active surface area (ECSA) with the number of cycles demonstrates the significant impact of the electrode fabrication method and catalyst loading on the catalyst durability with considerable ECSA values for electrosprayed electrodes. Electrodes prepared with low platinum loadings (0.05 mg Pt/cm^2^) exhibit elevated catalyst activity and stability under sulfuric acid conditions and maintain a crucial current density after 5 h of electrolysis. This work extends the understanding of the SO_2_-depolarized electrolysis (SDE) process and gives suggestions for further improvements in the catalyst layer fabrication, which provides potential support for the large-scale research and application of the HyS cycle.

## 1 Introduction

With the increase of global population, industrialization, and urbanization, energy demand is rapidly increasing, which contributes to environmental (global warming) and economic issues. In the light of this global warming and the negative consequences of the dependence on the conventional fossil fuel resources, the use of renewable systems, such as solar and wind energy, has significantly been escalating over the last few decades. However, these renewable energy sources pose reliability concerns due to their intermittent nature, causing a mismatch between the energy supply and demand.^1^ Thus, hydrogen is rising as an alternative and promising solution that has the potential to provide a clean, efficient, reliable, and affordable solution to improve the efficiency of energy utilization and to reduce greenhouse emissions.^2^ It was reported that hydrogen could meet 18% of the final energy demand, reduce 6 Gt of CO_2_ emissions annually, and create 30 million new jobs by 2050. Also, the literature shows that hydrogen could power over 400 million cars, 15–20 million trucks, and around 5 million buses in 2050, which is about 20–25% of the transportation industry.^3,4^

Green hydrogen can be produced in different ways by electrical, thermal, photonic, biological, and hybrid methods.^4−6^ At present, water electrolysis is considered as an optimal approach to produce pure and green hydrogen in a fast single-step process.^7^ However, the high-energy requirement is a major limitation of this process due to the large extent of the oxygen evolution reaction (OER). The OER contains multiple proton-coupled electron-transfer steps, which results in slow kinetics and a high activation energy barrier for O–O bond formation, which causes significant energy losses. Thus, many studies were established to decrease the energy consumption of this system by developing more efficient anodic electrocatalysts to reduce the overpotential of the OER^8−10^ or by replacing the OER with the oxidation of other species that are more easily oxidized than water.

Hence, the hybrid sulfur (HyS) cycle (also known as the Westinghouse cycle) has received much attention as a possible means for the efficient large-scale production of clean hydrogen.^11,12^ The HyS cycle is a two-step thermochemical/electrochemical process with a high-temperature thermochemical step (>850 °C) to dissociate sulfuric acid (H_2_SO_4_) into sulfur dioxide (SO_2_) and oxygen (O_2_) (Reaction 1) and a low-temperature electrolysis step (80 °C) to oxidize the SO_2_ in the anode of the electrolyzer (Reaction 2) and produce hydrogen in the cathode (Reaction 3).

**Figure 1:**
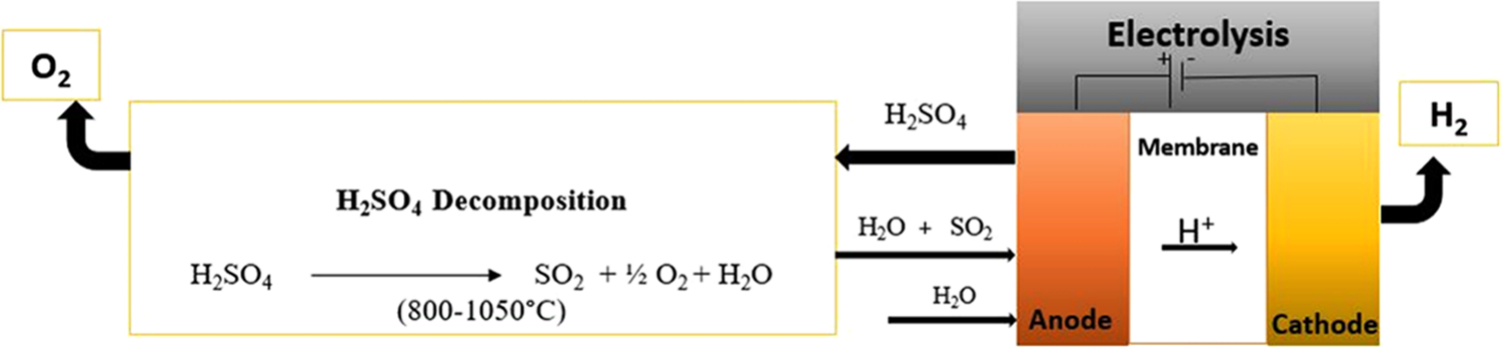
Scheme of the hybrid sulfur process.

This cycle needs about 55–80 kJ electrical power per mol of hydrogen with 180 kJ/mol of thermal energy.^13^ A schematic of the HyS process is shown in Figure 1.
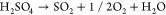
1
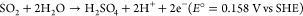
2
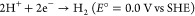
3Several studies have been established to improve the performance and the efficiency of the SO_2_-depolarized electrolysis (SDE) by developing new SDE electrolyzer materials and optimizing the operating conditions. Gorensek et al.^11,14,15^ extensively studied the thermodynamic properties and the operating conditions of this system using Aspen Plus. They determined the effect of sulfuric acid concentration on SO_2_ solubility and on the operating overpotential and discussed the interactions among the inlet flow rates, applied current, cell temperature, and pressure on the resulting cell voltage and acid concentration in the liquid product stream. Recently, it was concluded that constant current densities of 0.5, 0.75, and 1 A/cm^2^ can be produced at cell voltages of 0.59, 0.63, and 0.67 V, respectively, with an acid concentration of 65 wt % H_2_SO_4_, using a sulfonated polybenzimidazole (s-PBI) membrane and a platinum catalyst and by maintaining a cell temperature of 130 °C, a pressure of 2 bar, and a water-to-SO_2_ stoichiometry of 2.77.^15^ It is important to take into account that these results are not experimental but simulations with ideal conditions.

Krüger et al.^16−18^ investigated the pretreatment and stability of a PBI-based blended proton exchange membrane and the influence of membrane electrode assembly (MEA) manufacturing techniques such as hot-pressing on an SO_2_ electrolyzer. Also, they studied the effect of hydrogen sulfide H_2_S on the SDE.

It is well known that the SO_2_-depolarized electrolysis of water requires the use of electrocatalysts that plays a critical role to achieve high performance and to improve the anodic efficiency of sulfur dioxide oxidation. Although a broad range of catalysts have been developed for the anodic side,^19−21^ platinum-based electrocatalysts are still the most dominant catalysts used for both electrodes due to their high electrocatalytic activity and inert character.^22,23^

Colón-Mercado and Hobbs^12^ studied the catalytic activity and stability of Pt/C and Pd/C in different concentrations of sulfuric acid and at different temperatures. It was found that Pt/C provided better stability and activity than Pd/C, which was much less stable and less active for the oxidation of SO_2_. Also, the results showed that in very high sulfuric acid concentrations and at temperatures of 50 °C and above, Pt/C did exhibit instability.

Meekins et al.^24^ investigated the performance of thin films of commercial carbon-supported platinum and gold catalysts for SDE in ex situ (rotating disk electrode RDE) and in situ (single fuel cell) systems. It was demonstrated that Au can be used as a substitute catalyst for Pt for SO_2_ electrooxidation. Also, it was a more active and stable catalyst than Pt, Pd, and Ir.

Lee et al.^25^ studied the effect of platinum loading on SO_2_ oxidation of Pt/C electrodes with Pt loading amounts of 0.40, 0.80, 1.30, 2.34, and 4.02 mg/cm^2^. It was proved that the increase in the Pt loading from 0.4 to 4.0 mg/cm^2^ improved the SO_2_ oxidation. Hence, for a cell potential at 0.6 V, the apparent current density increased from 6.3 × 10^–3^ to 1.4 × 10^–2^ A/cm^2^ with an increasing Pt loading amount. However, the current density remained almost constant at a high potential for the five studied catalyst loadings, indicating that the SO_2_ oxidation reaction is controlled by the diffusion of dissolved SO_2_ to the electrode in the high anodic overpotential range.

Other works focused on preparing different types of bimetallic-electrocatalysts^22,26,27^ and demetallized catalyst^23^ as well as on improving the catalyst support.^28^ In our group, Pt-based catalysts have been successfully prepared and characterized with novel SiC/TiC supports. The highest activity and stability were found for these new electrocatalysts. In addition, the influence of temperature at values higher than 100 °C was evaluated using phosphoric acid-doped PBI-based membranes for the SDE.^29^

For fuel cell applications, the high cost and the limited sources of platinum are a significant barrier for the widespread use of these systems. Therefore, many researchers have been dedicated to reduce the Pt loading without sacrificing its performance by employing Pt alloys or by improving the fabrication techniques of the catalytic layer. The deposition techniques of the catalytic layer must be carefully selected to maintain the proton, electron, and gas transport properties and allow the coating of a uniform active layer. In the literature, different catalytic layer deposition techniques were developed to apply Pt-based electrocatalysts onto the membrane or the gas diffusion layers (GDL) to prepare membrane electrode assemblies (MEAs) with low metal loadings.^30−43^ A detailed review focused in this topic can be found elsewhere.^44^

To our knowledge, there are no studies that investigated the influence of the catalyst deposition technique on the activity and stability of the electrocatalyst in the HyS cycle. Hence, it is relevant to understand the critical role of the deposition method in cell performance.

In the current study, two different catalyst deposition techniques have been evaluated, the standard method (air gun, AG) and the advanced electrospray (ES) method, to fabricate electrodes with very low platinum loadings (in the range of 0.025–0.3 mg_Pt_/cm^2^, very low values obtained for the first time to the best our knowledge). Physical and electrochemical characterizations were carried out to determine the structure and performance of the prepared electrodes in the SDE system using the two different deposition techniques. Moreover, the stability of the catalyst layers (CLs) was also assessed.

## 2 Experimental Section

### 2.1 Catalytic Ink Formulation

The catalytic ink for air gun and electrospray deposition was prepared by mixing 65 mg of Pt/C powder (40% Platinum on Vulcan XC 72R (Carbon) from fuel cell store) and 83.5 mg of polybenzimidazole ionomer (1.98 wt % PBI in *N*,*N*-dimethylacetamide, DMAc) in DMAc as a solvent (10 mL). The mass fraction of PBI accounts for about 1/20 of the mass of the catalyst support. A small amount of PBI ionomer was added to improve the adhesion of the film to the electrode surface and to improve the proton conductivity. DMAc was added to adjust the viscosity and surface tension of the catalyst ink and to maintain the dissolution of PBI. Before spraying, the catalyst suspension was stirred in an ultrasonic bath for 1 h to ensure sufficient dispersion of the catalyst particles.

### 2.2 Electrospray Setup and Deposition of the Catalyst Layer on the GDL

Catalyst layers were deposited onto a gas diffusion layer (Freudenberg H23C2)-based carbon fiber paper coated with a carbon microporous layer (MPL).

A commercial electrospray device (Bioinicia, Spain) was used for the fabrication of the gas diffusion electrode (GDE).

The working parameters of the electrospray deposition were adjusted to maintain the Taylor cone jet mode. The flow rate was 800 μL/h, the voltage was 6 kV, and the distance between the needle and the substrate was 2 cm. These values were first optimized to achieve a homogeneous coating and to avoid a solvent-flooded catalytic layer that leads to undesirable cracks after solvent evaporation. Different gas diffusion electrodes (GDEs) were prepared by the electrospray method and the air gun spray technique with different Pt loadings in the range of 0.025–0.3 mg_Pt_/cm^2^ (these values are nominal ones, and the variation with the actual one was lower than 3.5% in all cases and the real ones were also taken into account for the different calculations). The surface area for the working electrode was 0.7068 cm^2^ cut from the original electrode (4 × 4 cm^2^) spread.

### 2.3 Physical and Electrochemical Characterizations of GDEs

The crystal structure of Pt catalyst in the layer deposited on working electrodes was analyzed by X-ray diffraction (XRD) in a Philips X’Pert MPD (PANALYTICAL, United Kingdom) diffractometer equipped with Cu Kα radiation. XRD analyses were carried out recording the 2θ angular region from 10 to 100° (scan rate, 0.02°/s). The platinum crystallite size *L*_C_ is determined using the Scherrer equation (eq 4)
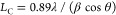
4

where *L*_C_ is the crystal size in nm, λ corresponds to the Kα radiation of copper (λ = 0.15418 nm), β is a parameter related to the full width at half-maximum intensity of the peak, and θ is the angle corresponding to the maximum intensity (rad).

The surface morphology of Pt/C electrodes was examined by field emission scanning electron microscopy, SEM (Gemini SEM 500 field emission microscope), and energy-dispersive X-ray spectroscopy (EDS).

**Figure 2:**
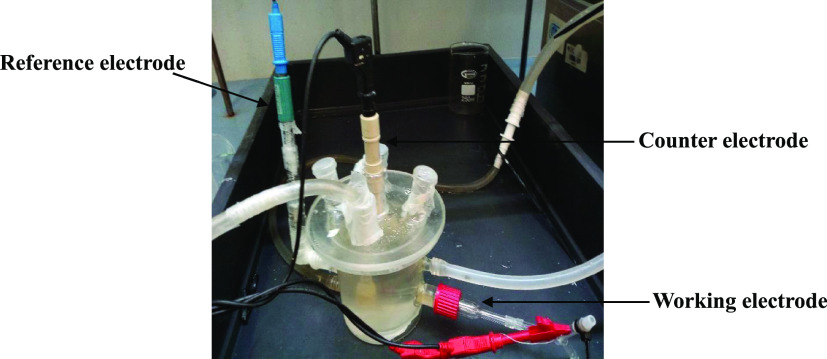
Half-cell setup for electrochemical characterization.

The durability of catalyst layers created on the GDEs was characterized in a half-cell setup described elsewhere.^28^ The electrochemical surface-active area (ECSA) was investigated by cyclic voltammetry (CV) in a typical three electrochemical cell system using Ag/AgCl (KCl 3M) as the reference electrode, a platinum wire as the counter electrode, and the prepared GDE as the working electrode in a deaerated solution of 1 M H_2_SO_4_ as the supporting electrolyte at 50 °C (Figure 2). The GDE was directly contacted with a gold piece that assembled directly with the working electrode, and two gaskets were used to avoid the penetration of electrolyte in the electrode assembly and to ensure the good contact. CVs were performed from 0.0 to 1.2 V vs reversible hydrogen electrode (RHE) for 500 cycles at a scan rate of 50 mV/s using a galvanostat/potentiostat (AutoLab PGSTAT204, the Netherlands).

The ECSA was calculated according to eq 5. It provides important information regarding the number of available active sites. The ECSA (m^2^/g_Pt_) was obtained from the charge required for hydrogen adsorption–desorption from the Pt electrocatalyst.
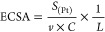
5where *S*_(Pt)_ is the surface under the hydrogen desorption peak (AV/cm^2^), *v* is the scan rate (V/s), *C* is a constant related to the required load to reduce the proton layer on the active platinum (0.21 mC/cm^2^), and *L* is the platinum load in the catalyst layer (mg_Pt_/cm^2^).

A chronoamperometry study was carried out to evaluate the stability and SO_2_ oxidation efficiency electrolysis of the different electrodes in a half-cell setup for 5 h. Taking into account that the electrode area was too small (0.7068 cm^2^) and the Pt loadings to be tested are also very low, the SO_2_ consumption is expected to be low, and for this reason, SO_2_ was bubbled for 1 min as in a previous work.^28^ A potential of 1.0 V was applied for all of the experiments.

Hence, using Faraday’s law of electrolysis, the theoretical mass of produced hydrogen is calculated using eq 6
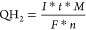
6where *I* is the current (in Amps) that passes through the cell during a period *t* (in second), *M* is the molar mass of H_2_, *F* is the Faraday constant (96 485.33 C/mol), and *n* is the number of electrons, which is 2 in the SDE system.

CVs and linear sweep voltammetries (LSVs) were performed before and after the chronoamperometry test (LSVs were carried out in 1 M H_2_SO_4_ solution between 0.2 and 1.2 V vs RHE at 50 mV/s and at 25 °C). Moreover, electrochemical impedance spectroscopy (EIS) was also performed to evaluate the ohmic and charge transfer resistances of the prepared electrodes. EIS was conducted at 0.6 V vs RHE with an amplitude of sinusoidal interference signal of 5 mV, and the frequency range is 0.1 to 10.000 Hz.

## 3 Results and Discussion

### 3.1 Morphology of Catalyst Layers

Physicochemical characterization of the GDEs was performed to compare the influence of the deposition technique and platinum loading on the structure of the catalyst layers (CLs).

**Figure 3:**
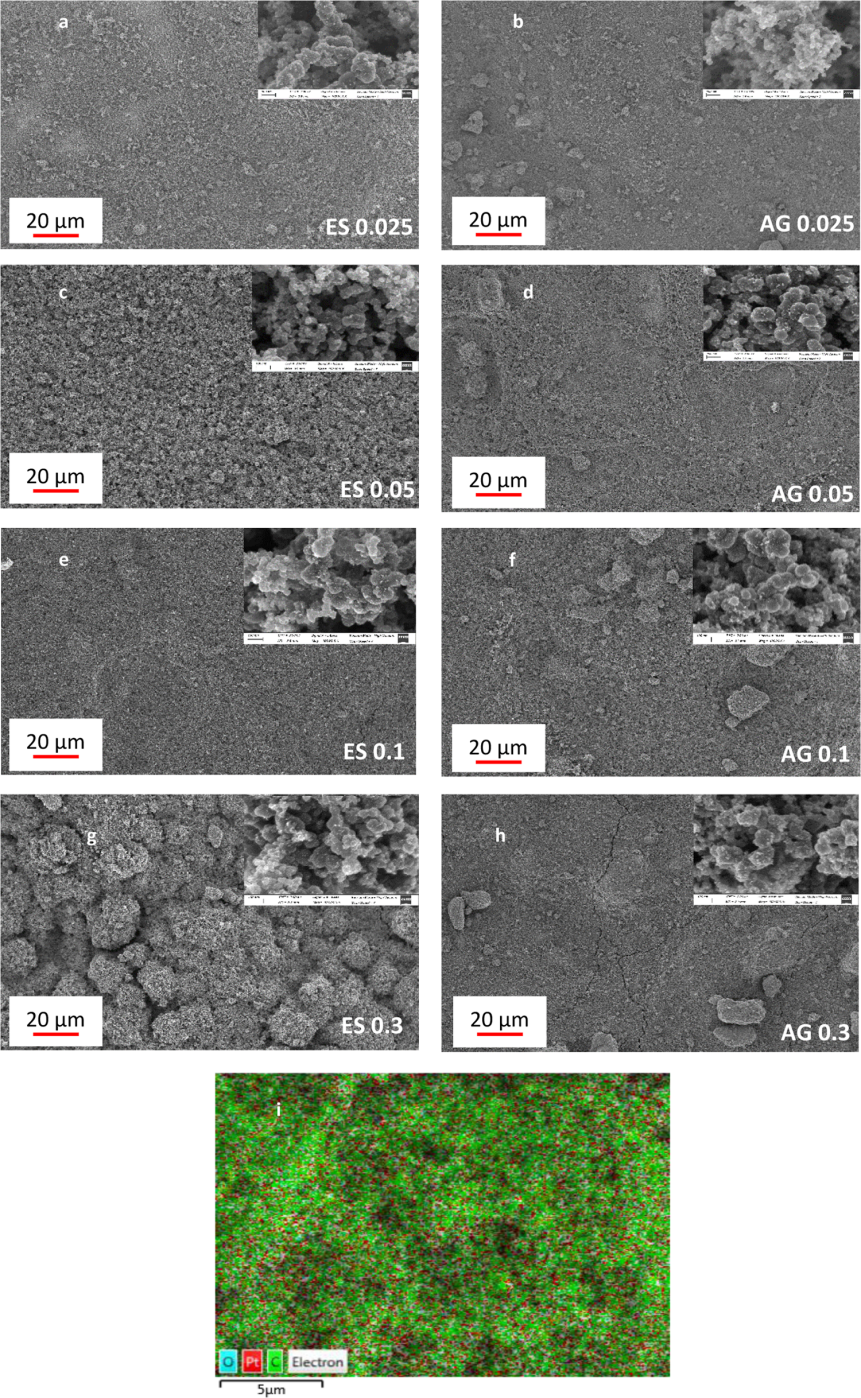
(a–h) SEM images of the catalyst layers prepared with electrospray (ES) and air gun (AG) with different platinum loadings. (i) EDS image of the surface of the catalyst layer.

Figure 3a–h shows SEM images of the surface morphology of deposited layers by air gun and electrospray on the carbon paper electrodes for different loadings of 0.025, 0.05, 0.1, and 0.3 mg_Pt_/cm^2^. A great difference is detected in the microstructure surface of the different GDEs.

It is well known that the mechanism of the electrospray technique creates a good dissociation of most of Pt/C aggregates into single aggregates, and it is able to disperse the ionomer phase within the mesoporous structure of the catalyst layer. Also, in the electrospray deposition, the solvent is expected to dry in flight and only a relatively dry solid would collide into the substrate causing a thicker and less compact layer.^45,46^ For these reasons, when the catalyst loading was 0.025 mg_Pt_/cm^2^, the ink was dispersed over the MPL and cover only the surface of the individual fiber of the GDL. By increasing the catalyst loading and adding more catalyst ink (more layers of the electrosprayed ink over the substrate), the catalyst layer becomes more defined and continuous, and progressively covers the total surface of the GDL. This observation was also confirmed by Martin et al.^47^

The surface of the electrosprayed catalyst layers shows a highly uniform morphology without aggregates typically observed on the surface of CLs prepared by air gun, which may decrease the number of Pt particles participating in the reaction. For electrodes prepared with air gun, the catalytic layers appear dense and less porous with Pt/C aggregates covering a wider range of sizes and cracking was observed for AG 0.3 that may decrease the ionic and electronic transfer. On the contrary, a homogeneous layer of particles is observed for electrosprayed GDEs. This behavior is continuous in the entire electrode surface and exhibits a porous CL along the GDL, especially for ES 0.05, which improves the access of reactants to the platinum particles and consequently leads to better Pt utilization.

For the nanoscale (subgraphs), the surface of catalyst layers is composed of platinum nanoparticles (NPs), which appeared as bright dots, supported on spherical carbon black and distributed on the surface of the MPL. The diameter of the carbon black ranges from 20 to 50 nm. CLs exhibited a very porous architecture and homogeneous agglomeration of carbon for all electrodes.

The appearances of the surface of electrodes prepared with the two methods are similar except that the GDE was fabricated with air gun with 0.025 mg_Pt_/cm^2^ as Pt loading, where the size of the carbon black is smaller, and the distribution of Pt nanoparticles is not well defined. Clearly, the Pt nanoparticles (1–4 nm) are uniformly dispersed, resulting in a homogeneous layer along the GDL, and some small clusters are also observed in Figure 3d–h when the platinum loading increased and for electrodes sprayed with air gun. However, for electrosprayed electrodes with low loadings (0.025 and 0.05 mg_Pt_/cm^2^), Pt catalyst nanoparticles (NPs) have a very narrow size distribution (with no clusters). Therefore, the electrospraying technique could reduce agglomeration of the carbon-supported platinum particles, which is a great advantage for the formation of the triple-phase boundary (TPB) and consequently beneficial to improve the Pt utilization. The CL surface was also characterized by EDS (Figure 3i), from which the platinum showed a uniform distribution throughout the entirety of the studied area. This observation is consistent with previous works that show similar morphology and distribution for catalyst layers prepared with electrospray and air gun.^46−50^

**Figure 4:**
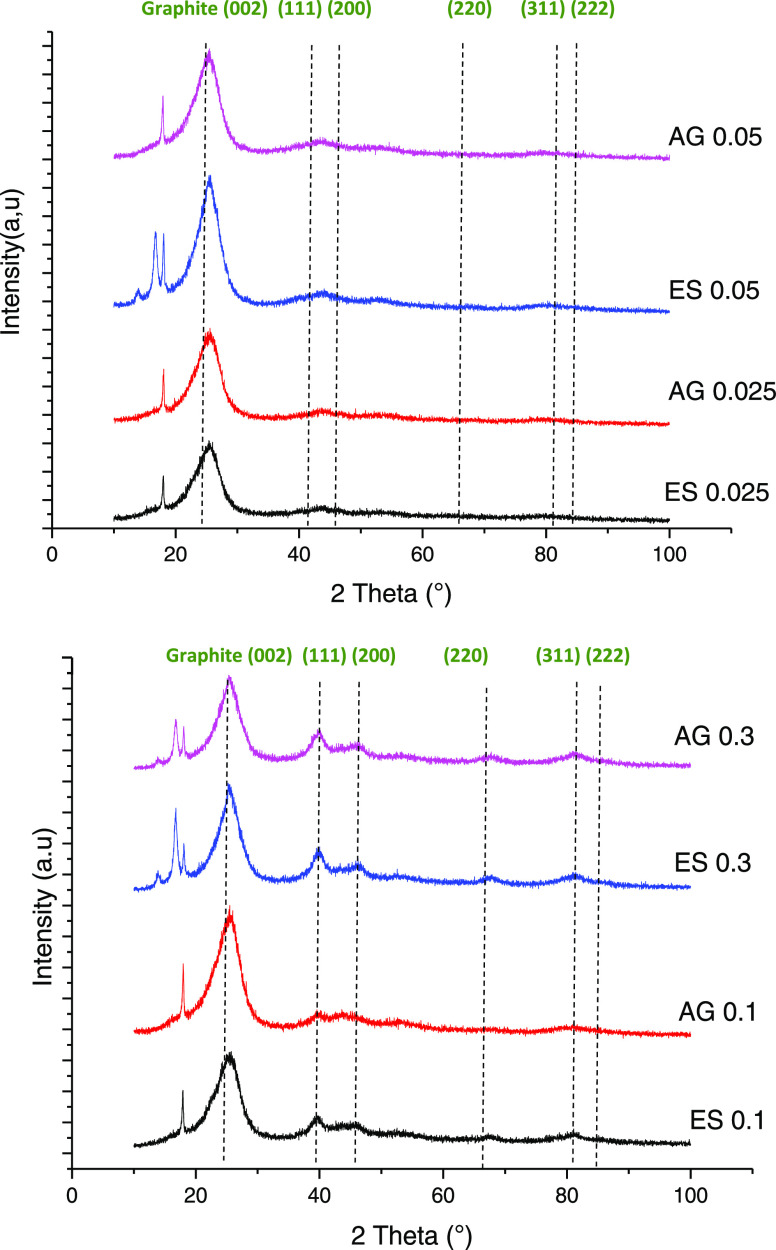
XRD pattern of the pristine GDEs.

Figure 4 depicts the XRD pattern of the pristine Pt/C catalyst layers. The diffraction peaks located at 2θ = 25.5° belong to the carbon support. For ES 0.3 and AG 0.3 electrodes, two peaks of crystalline species were observed at 2θ = 39° and 81°, corresponding to the (111) and (311) planes of face cubic centered (fcc) polycrystalline Pt.^28^ The intensity of the peak corresponding to the Pt(111) plane was much higher than that of the Pt(311) plane, which indicated that the (111) direction is the predominant growth direction of the Pt crystal. For ES 0.1 and AG 0.1, one peak with a low intensity was obtained corresponding to the Pt(111). Furthermore, no diffraction peak of platinum was observed in the XRD spectra of ES 0.025, ES 0.05, AG 0.025, and AG 0.05 electrodes, which can be due to the low amount of Pt in the CL or due to the better distribution on the surface.^51^

### 3.2 ECSA Variation of Prepared GDE

**Figure 5:**
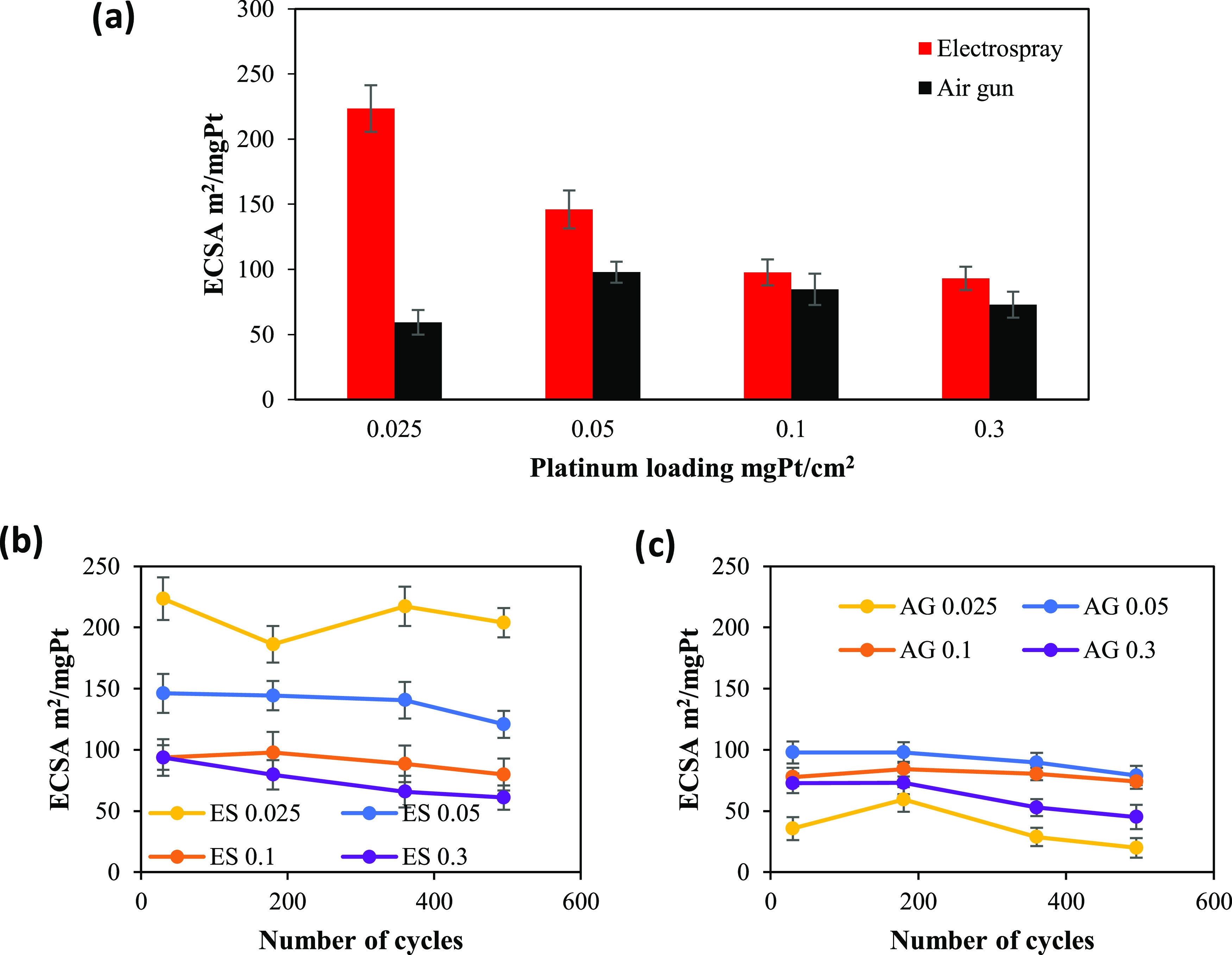
(a) Maximum ECSA values obtained for the prepared GDEs with different platinum loadings. (b, c) Durability of the Pt/C electrodes prepared with (ES) electrospray and (AG) air gun during 500 cycles in nitrogen-saturated 1 M H_2_SO_4_.

Electrochemical active surface area (ECSA) is one of the most important characteristics of Pt/C electrocatalysts. Generally, the value of ECSA is related to the triple-phase boundary (TPB) and provides important information regarding the number of available active sites in the catalyst layer. Hence, to compare the effect of the deposition technique on the performance and durability of the catalyst layer, CVs analyses were performed in a N_2_-saturated 1 M H_2_SO_4_ electrolyte solution for different GDEs prepared with air gun and electrospray, and the ECSA values were calculated. Figure 5 shows the values of ECSA obtained by the electrodes prepared with the two different deposition techniques studied in this work and with different Pt loadings.

Using eq 5in the Experimental Section, the ECSA values of the sprayed and electrosprayed working electrodes were calculated and compared.

Figure 5a shows the maximum ECSA values obtained for the various platinum loadings. As observed, the ECSA values for GDEs prepared with electrospray are higher in all cases compared with the air gun deposition method. In addition, a remarkable difference of maximum ECSA values between the two techniques was obtained for electrodes prepared with 0.025 and 0.05 mg_Pt_/cm^2^, when the catalyst loading is very low. Also, the activity of the catalyst layer increased with the decrease of the platinum loading except for the electrode AG 0.025 when the ECSA was lower than AG 0.05. At high Pt loadings (e.g., 0.1 and 0.3 mg/cm^2^), electrodes prepared with air gun and electrospray yield comparable ECSA. It must be highlighted that the obtained values by the AG electrodes agree with the values obtained by our group in a previous work,^28^ where using the same experimental procedure and the same commercial Pt-based catalyst, an ECSA value of 45.1 m^2^/g_Pt_ was achieved for an electrode prepared with air gun and with a Pt loading of 0.5 mg Pt/cm^2^. However, when the Pt loading is decreased to 0.05 and 0.025 mg/cm^2^, the maximum ECSA decreased by 70% from ES 0.025 to AG 0.025 electrodes. It can be observed that the electrospray method provides better performances compared to the air gun method, especially at low platinum loadings. A possible explanation for our findings is due to the fact that electrospray leads to a better distribution of the catalyst ink onto the GDL and homogeneous material penetration (as observed by SEM images) into the substrate during the catalyst deposition giving better platinum utilization. These experimental results are consistent with previous results.^46,49,52^

In fact, the deposition technique and the type of substrate influence the porosity of the catalytic layer and the degree of penetration of the catalyst into the selected substrate. Hence, to characterize the TPB, the primary parameters that should be taken into consideration are the pore size distribution, porosity, and tortuosity in the CL structure. Martin et al.^53^ investigated the effect of catalyst deposition methods (ultrasonic spraying, airbrushing, and electrospraying) on the performance of a fuel cell. By characterizing the morphology of the surface microstructure of the different catalytic layers, it was reported that a granular-like catalytic layer with Pt/C aggregates covering a wider range of sizes was obtained with airbrush spraying and with a porosity of less than 50%. Electrodes prepared with ultrasonic spray coating exhibit a smooth surface dotted with a number of Pt/C aggregates (ca. 50–80 mm size). On the contrary, a porosity of more than 70% with a homogeneous distribution of pores sizes was observed for electrosprayed electrodes, which favors TPB creation. Consequently, the highest performance is obtained by the electrosprayed MEA. In another work,^46^ the influence of air gun and electrospray deposition on the morphology of the CL was studied. A dendritic morphology was observed for electrodes fabricated by electrospray deposition, with enhanced meso- and microporosities. Also, it was demonstrated that the porosity increases on electrosprayed films, whereas it decreases on airbrushing with aggregate formation. Hence, the highest ECSA (44 m^2^/g_Pt_) was obtained for the highest-porosity film prepared with the electrospray technique.

Úbeda et al.^54^ reported the same results. A significant performance enhancement (by about 40%) was obtained using the electrospray deposition to prepare the catalyst layers of a PBI-based high-temperature PEMFC. This improvement in cell performance was due to the enhanced mass transport and higher ECSA (better catalyst utilization).^44^

ECSA durability is considered an important parameter to the practical application of catalyst electrodes. Thus, Figure 5b,c reports the evolution of ECSA with the number of cycle numbers for the two deposition techniques. As seen, during the 500 cycles, the ECSA values of electrosprayed GDEs are higher than those for the GDEs prepared with air gun. For the catalyst layers prepared with electrospray, the ECSA lines follow an inverse relation with the platinum loading: the highest ECSA was obtained for the lowest Pt loading. However, for electrodes fabricated with air gun, the low-level line was attributed to AG 0.025. The ECSA evolution for ES 0.025 and AG 0.025 is not stable, and a rapid decrease rate was observed for the two electrodes despite the higher values of ES 0.025. These data could be due to the effect of the deposition method on the dispersion of catalyst particles on the surface of the GDL. By comparing the ES 0.05 and AG 0.05, the ECSA values in both cells decreased at a lower rate for the 200 initial cycles and it appeared to stabilize at ∼140 and 100 m^2^/g for ES 0.05 and AG 0.05, respectively. After the 200 cycles, ES 0.05 exhibit a smaller drop in ECSA until 400 cycles. However, for AG 0.05, a significant decrease was observed after 200 cycles. For ES 0.1 and AG 0.1, during the first 100 cycles, the two electrodes exhibited an increase in the ECSA. This gain in ECSA can be linked to a temporary enhancement in Pt dispersion by Pt surface atoms or Pt particle rearrangement. After 100 cycles, AG 0.1 appeared to be more stable compared to ES 0.1. For electrodes with a high Pt loading (0.3 mg/cm^2^), the ECSA decreased markedly after 200 cycles. According to Figure 5 and Table 1, the Pt/C electrosprayed electrode demonstrates good stability and activity, whereas for sprayed electrodes, a significant loss was obtained, especially with low Pt loadings. This can be attributed to a decrease in the particle size of the catalyst with the application of spraying voltage, which is beneficial for generating a three-phase reaction interface between the carbon support, the binder, and the platinum particles, in which the proton and electron conductivities of the catalysts can be effectively improved.

**Table 1:** Specific Values Associated with Electrochemical Characterization for Different GDEs

electrodes	ECSA drop after 500 cycles (%)	maximum current density for SO_2_ oxidation (mA/cm^2^)^a^	*E*_onset_ (V)^a^	overpotential (mV/dec)^a^	activity drop at 1 V (%)
ES 0.025	9.00	59	0.660	99.20	13
AG 0.025	49.50	56	0.662	102.30	11
ES 0.05	17.31	81	0.610	84.86	12
AG 0.05	19.30	72	0.620	90.75	8
ES 0.1	18.40	92	0.562	80.6	5
AG 0.1	13.60	100	0.580	82.45	9
ES 0.3	35.00	110	0.525	67.29	b
AG 0.3	38.10	130	0.570	70.33	1.5

a The maximum values of the current densities, *E*_onset_, reported in the table are from LSVs after 5 h.

b There is no activity drop for the ES 0.3 electrode.

The ECSA loss of Pt/C after potential cycling can be associated with the agglomeration of Pt particles, as evidenced XRD analyses represented in S1 (Supporting Information).

Particle growth is a typical consequence of Pt dissolution/redeposition (also known as Ostwald ripening) or coalescence via crystal migration.^55^ The size of Pt-based catalyst used in PEMFC is in the nanometer scale, usually in the range of 2–6 nm. Nanoparticles inherently show a strong tendency to agglomerate due to their high specific surface energy. For nanoparticles, the smaller the size, the higher the specific surface area and the easier the agglomeration/sintering. Hence, when Pt nanoparticles agglomerate to bigger ones, the electrochemical surface area of Pt catalysts decreases, and consequently, the performance of PEMFC degrades.^56,57^ Thus, the growth of Pt particles with aging was confirmed by XRD. The crystallite sizes of Pt(111) of pristine electrodes ES 0.3 and AG 0.3 were 2.7 and 2.9 nm, which increased to 5.8 and 6 nm, respectively, after 500 cycles. For ES 0.1 and AG 0.1, it was 3.4 and 3.9 nm before cycling and 21.7 and 11.7 nm after ECSA durability, respectively. The Pt(111) peak is not observed for ES 0.025, ES 0.05, AG 0.025, and AG 0.05 after 500 cycles. The absence of this peak could be due to the low loading of the platinum catalyst.

### 3.3 Catalytic Activity for SO_2_ Oxidation Reaction

**Figure 6:**
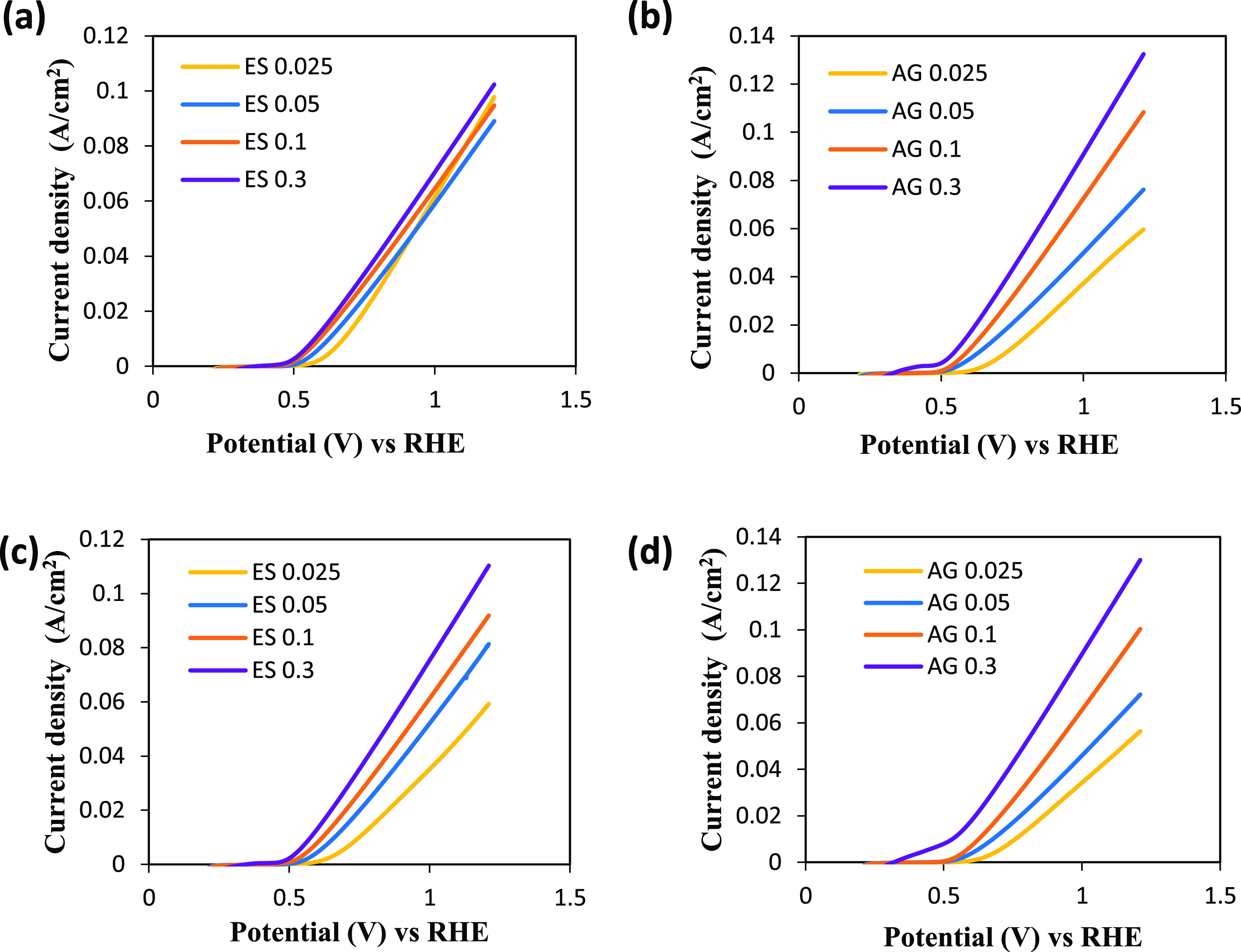
LSVs of Pt/C electrodes prepared with electrospray and air gun, in SO_2_-bubbled 1 M H_2_SO_4_ solution between 0.2 and 1.2 V at 50 mV/s (a, b) before and (c, d) after chronoamperometry characterization.

The electrocatalytic activity of the Pt/C catalysts deposited onto GDLs by electrospray and air gun with different platinum loadings was investigated for the oxidation of SO_2_ in sulfuric acid solutions for hydrogen production. Figure 6 displays the LSV plots for anodic SO_2_ oxidation before and after 5 h of chronoamperometry test for the electrodes prepared with the two different techniques. According to a previous work,^20^ it was reported that sulfur species formed at low potentials could either enhance or inhibit the reaction, depending on the extent of the surface coverage. The operation conditions used in this test could produce sulfur species, but this fact would make that this essay could be considered as an accelerated stress test to avoid long test periods and the results are reliable.

The onset potential (*E*_onset_) was calculated using the intercept of two straight lines tangent to the linear polarization curve and is reported in Table 1. As the Pt loading increased from 0.025 to 0.3 mg/cm^2^, *E*_onset_ decreased from 0.660 to 0.525 V vs RHE and from 0.662 to 0.570 V vs RHE for GDEs prepared by electrospray and air gun, respectively. Meanwhile, the current densities increased with the increase of Pt loading for both techniques and in the whole range of voltage studied. This trend is the same that Lee et al.^25^ obtained when they studied the effect of Pt loading for the SDE reaction. They carried out LSVs from 0.4 to 1.4 *V*_RHE_ to electrodes prepared by brushing and in a SO_2_-saturated 50 wt % H_2_SO_4_ solution with Pt loading from 0.4 to 4.02 mg Pt/cm^2^. The current densities were independent of the Pt loading at voltages higher than 0.8 V, and the current density at that voltage was around 0.04 A/cm^2^. However, at lower voltages, the influence of Pt loading could be observed. Values from around 0.005 to 0.01 A/cm^2^ at 0.6 V from the lowest to highest Pt loading, respectively, were reached. In the present work, at 0.6 V, the current densities of ES 0.3 and AG 0.3 (0.3 mg/cm^2^ as platinum loading) are twice with 0.012 and 0.017 A/cm^2^, respectively. Thus, according to this observation, it could be confirmed that the deposition method has a great influence on the electrochemical activity toward the SO_2_ oxidation of the catalyst layer.

**Figure 7:**
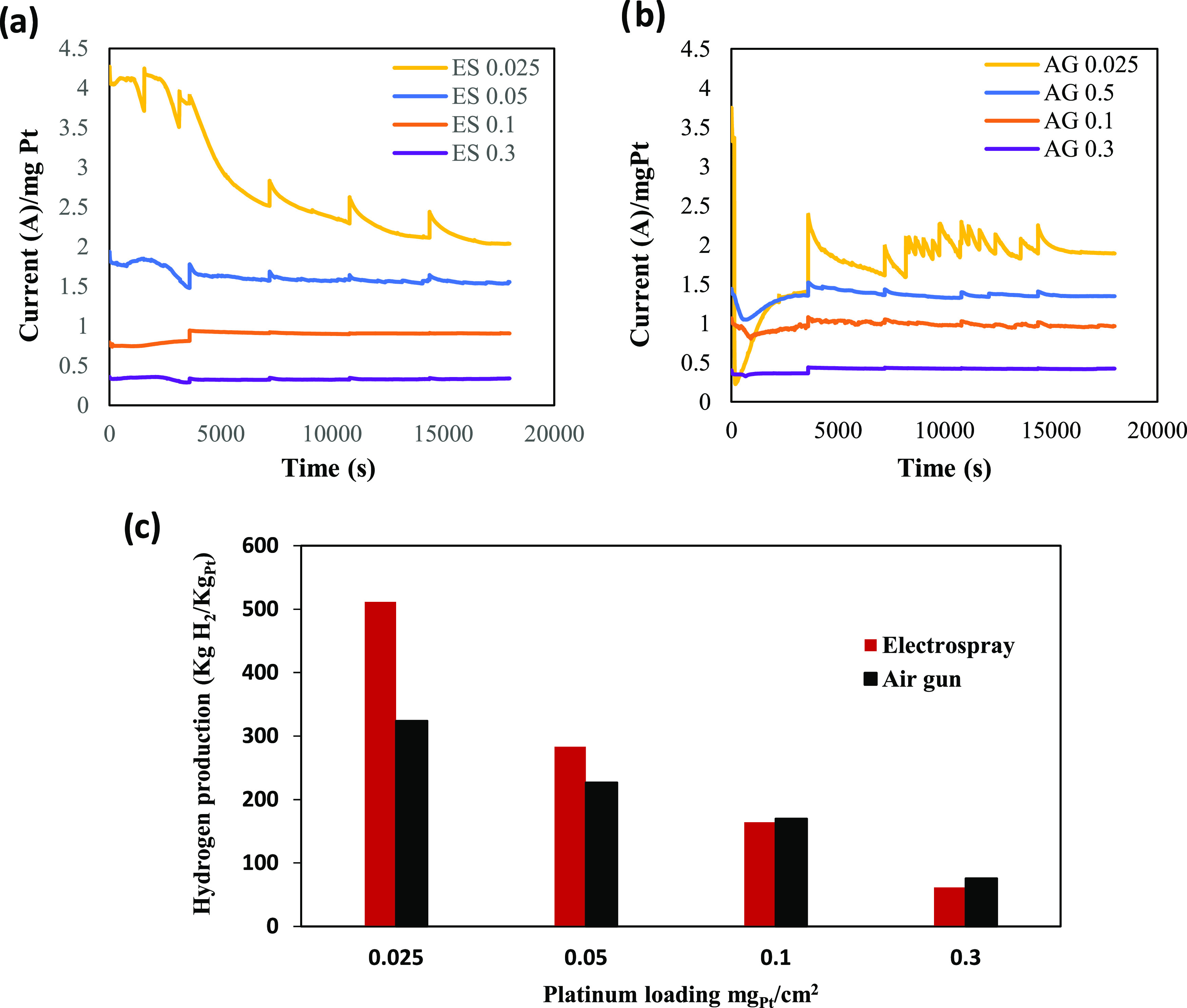
(a, b) Chronoamperograms of electrodes in SO_2_-bubbled 1 M H_2_SO_4_ electrolyte for SO_2_ oxidation at 1 V. (c) Theoretical hydrogen production for different electrodes during 5 h of electrolysis.

The electrochemical stability of the catalyst layer and SO_2_ oxidation efficiency were tested by carrying out a chronoamperometry test in a half-cell system. For all of the experiments, a potential of 1.0 V was applied. The results are displayed in Figure 7.

At the beginning of the experiment (Figure 7a,b), for GDEs prepared with air gun, the current decreases to a larger value, which then rapidly increases and tends to be stable. For catalyst layers deposited by electrospray, the current decreases with a slow rate during the first record except the ES 0.1 electrode. After the first hour, the current of electrodes prepared with 0.05, 0.1, and 0.3 mg/cm^2^ for both techniques are comparatively stable and no significant changes were found during the experiment. However, for ultralow Pt loading, Pt/C presented a fast decrease of current for ES 0.025 and an unstable activity for AG 0.025. In fact, the distribution of a low amount of catalyst resulted in a very thin layer, where the degradation effects of this stress test are observed rapidly. Hence, a deeper optimization of the electrospray technique can be performed for an optimal catalytic layer deposition for such a low Pt loading.

By comparing the maximum current densities for the different electrodes and as reported in Tables 1 and S2, it can be seen that for the two highest loadings (0.1 and 0.3 mg/cm^2^), the current densities obtained for GDEs prepared with air gun are relatively higher than GDEs fabricated with electrospray. However, at the end of the experiment, the current density decreases by about 5% for ES 0.1 and by 9% for AG 0.1. For ES 0.3, a slow increase of the current by about 8% was obtained against a decrease by 1.5% for AG 0.3. For low loadings (0.025 and 0.05 mg/cm^2^), higher specific catalytic activity was obtained for electrosprayed electrodes. It should be highlighted that the current densities obtained in this work during the chronoamperometry test (see Figure S2) are higher than the values obtained by other authors such as Dourado et al.,^58^ where current densities below 0.03 A/cm^2^ were obtained for a disk Pt electrode in an H-shaped cell with a SO_2_-saturated 0.5 M H_2_SO_4_ solution.

Figure 7c reports the theoretical hydrogen production values obtained for the electrodes with different platinum loadings according to eq 6. As shown for the two lowest loadings, significant quantities of H_2_ were obtained for GDE prepared with electrospray layers that were higher than those of electrodes fabricated with the air gun deposition method.

It is important to mention that at the end of the chronoamperometry of SO_2_ oxidation, ES and AG electrodes show similar activity. However, the hydrogen production in the case of the ES electrodes was higher than in the case of AG electrodes for the two lowest Pt loadings. Furthermore, an evident enhancement is observed when the electrospray technique is used to prepare electrodes with very low catalyst loadings. At higher loadings, this effect is not observed. Therefore, this behavior is in line with what we want to point out. Electrospray is a very good candidate for very low catalyst loadings. Also, during the preparation of the electrode, the catalyst loss caused by air gun is higher than that caused by the electrospray technique because the trajectory is well defined for electrospray. This means that cost-effective electrodes can be prepared using the electrospray deposition technique, which will reduce the cost of hydrogen production using low platinum loadings.

As seen in Figure 7, the current densities interval from AG 0.05 to AG 0.3 is smaller than the gap from ES 0.05 to ES 0.3, which can confirm the influence of the catalyst layer deposition technique for electrodes with low loadings on the performance of the active sites of the catalyst layer. On the other hand, the performance at a loading of 0.1 mg/cm^2^ is not twice that observed at a loading of 0.05 mg/cm^2^. Consequently, extremely high performance cannot be easily obtained by just increasing the platinum loading. Hence, high stability and activity can be enhanced by improving the catalyst layer fabrication with low platinum loading. By comparing intensities before and after the chronoamperometry tests, the obtained current densities are very similar. The small reduction in intensity during these long experiments could be caused by catalyst agglomeration confirmed by XRD results.

ECSA characterization was carried out before and after chronoamperometry tests to estimate the active sites after SO_2_ oxidation (S3). For ES 0.025 and AG 0.025, no hydrogen adsorption/desorption peaks were observed after the oxidation experiment. For the other electrodes, ECSA values were decreased by about 50%. The decrease in ECSA could be related to the occupation of active sites by SO_2_ and intermediate products (S4) and the agglomeration of catalyst particles, thus changing the properties of Pt surface and decreasing adsorption/desorption of hydrogen.

For comparison purposes and to the best of our knowledge, the effect of catalyst deposition technique has not been studied for SO_2_ oxidation and even electrodes with such a low Pt loading have not been tested either for the SDE. In a previous study by our group,^28^ the stability of Pt/C deposited by air gun with a catalyst loading of 0.5 mg/cm^2^ was presented. By comparing the results, no significant difference can be observed between electrodes with a low Pt loading and electrodes prepared with 0.5 mg/cm^2^ as the AG electrodes (0.5 mg Pt/cm^2^) showed great stability after a chronoamperometry test of 14 h in a 1 M H_2_SO_4_ solution bubbled during 1 min with SO_2_.

**Figure 8:**
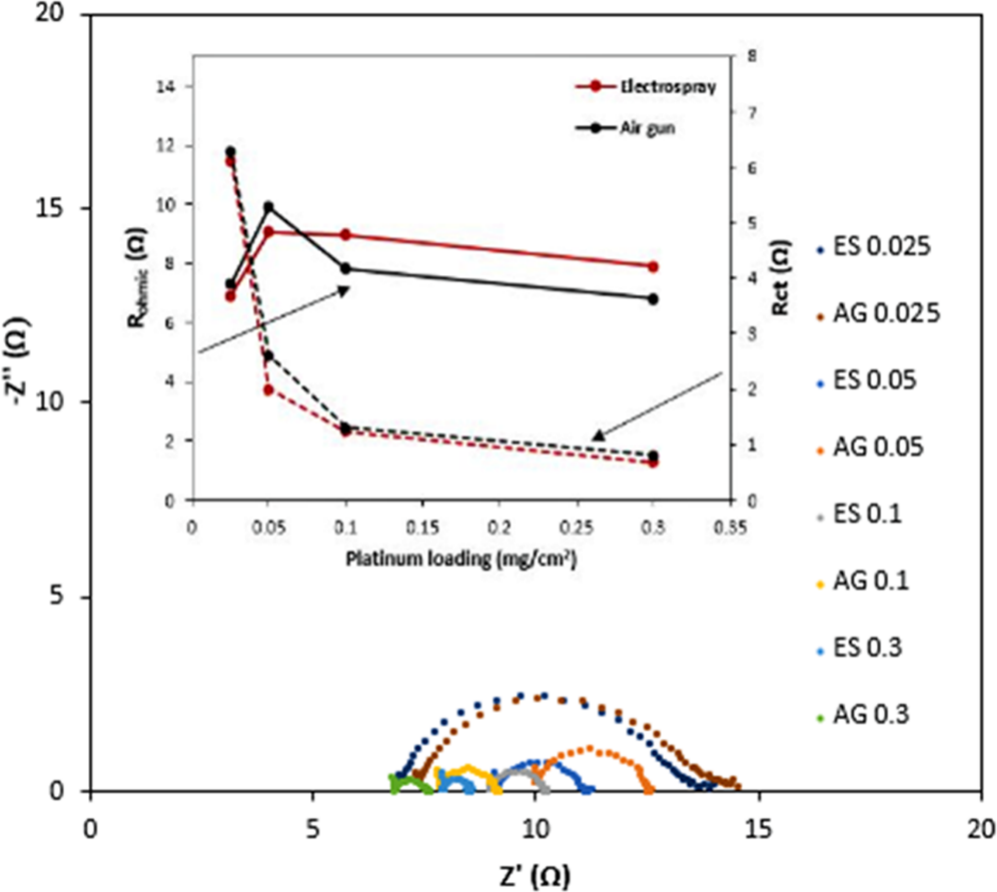
Nyquist diagram of electrodes in the working electrolyte at 0.6 V with subgraph of ohmic and charge transfer resistance variation.

After chronoamperometry tests, an electrochemical impedance spectroscopy (EIS) test was performed to investigate the catalytic activity of the catalysts for SO_2_ electrooxidation. The impedance performance of different GDEs in the half-cell system is expressed by Nyquist curves, as shown in Figure 8. The Nyquist diagram of each catalyst shows a semicircular shape. The intersection point of the curve at a low frequency and the horizontal axis reflects the total ohmic impedance (*R*_ohmic_) of the electrolysis system, and the diameter of the arc in the Nyquist curve indicates the charge transfer resistance (*R*_ct_) of the SO_2_ oxidation process. By fitting the equivalent circuit, the respective ohmic resistance and charge transfer resistance on the electrode can be calculated, and the specific variations are shown in the subgraph in Figure 8. As shown in Figure 8 and Table S1, the charge resistance transfer of SO_2_ electrooxidation decreases with the increase of Pt loading for both electrodes prepared by electrospray and air gun, indicating the fastest electrochemical reaction rate and the easiest charge transfer of reactants. Also, the *R*_ct_ value of electrosprayed electrodes was relatively lower than the resistance obtained for GDEs prepared with air gun. The higher ohmic resistance could be related to the resistance of the solution after SO_2_ oxidation. The relationship of *R*_ct_ values in each catalyst is consistent with the mass activity in the LSVs and the chronoamperometry tests (a higher charge transfer resistance means a lower charge transfer rate and a higher overpotential).

## 4 Conclusions

In summary, platinum nanoparticles have been successfully deposited onto gas diffusion layers by electrospray and air gun techniques to fabricate GDEs with several platinum loading amounts ranging from 0.025 to 0.3 mg/cm^2^. Comparison between electrodes was carried out to study the effect of Pt loading and the electrode fabrication method on SO_2_ oxidation reaction in an SDE process. We have demonstrated that the electrospray technique is a promising technique to prepare catalyst layers as it showed uniform catalyst distribution over the GDLs. Hence, the structure of the electrosprayed electrodes has the advantage of increasing the electrochemical surface area and enhancing the durability of the catalyst layer for GDEs prepared with low platinum loading. In addition, high SO_2_ catalytic oxidation activity and stability in the sulfuric acid electrolyte were found for electrodes with a platinum loading of 0.05 mg_Pt_/cm^2^ with current densities reaching 1.5 A/mg_Pt_, slightly higher for electrosprayed electrodes. At the low platinum loading, the H_2_ production per kg of Pt of the electrosprayed electrode was 37% higher than electrodes fabricated using the air gun technique. In the future, crucial work will be established in a single-cell setup to optimize this process and to evaluate the Pt content and the electrode fabrication method.

This study features a certain significance of the deposition method for high performance of the anode electrode with a relatively low noble-metal content, effectively reducing catalyst costs and promoting the application of the Westinghouse system for green hydrogen production.

## Supporting Information Available

XRD pattern of the GDEs after 500 cycles, chronoamperograms of electrodes in the working electrolyte for SO_2_ oxidation at 1 V for different Pt loadings, cyclic voltammetries of electrodes in nitrogen-saturated 1 M H_2_SO_4_ before and after SO_2_ oxidation for different Pt loadings, SEM images of the catalyst layers prepared with electrospray and air gun with different platinum loadings after SO_2_ stability test; and charge transfer resistance *R*_ct_ and ohmic resistance *R*_s_ data for the different GDEs after chronoamperometry tests (PDF)
