# Supplementary material for: Electrospray Deposition of Catalyst Layers with Ultralow Pt Loading for Cost-Effective H2 Production by SO2 Electrolysis
Source: ACS Appl Energy Mater. 2022 Feb 4;5(2):2138–49. doi: 10.1021/acsaem.1c03672 (PMC8889905; doi:10.1021/acsaem.1c03672)
Supplement: Supplementary file 1 — ae1c03672_si_001.pdf [file ae1c03672_si_001.pdf]

Supporting Information

Electrospray Deposition of Catalyst Layers With Ultra-Low Pt Loading for Cost Effective H_2 Production by SO_2 Electrolysis

Imen Fouzai [1,2], Maher Radaoui[1], Sergio Díaz-Abad[3], Manuel Andrés Rodrigo[3], Justo Lobato*[3]

[1] Laboratory of Technology, Energy, Materials and Innovation "TEMI, Faculty of Sciences of Gafsa, Cité Sidi Ahmed Zarroug 2112 Gafsa, TUNISIA

[2]National Institute of Applied Sciences and Technology, B.P. No 676, 1080 Tunis Cedex, TUNISIA

[3] Department of Chemical Engineering. Faculty of Chemical Sciences & Technologies. University of Castilla-La Mancha. Campus Universitario n12. 13071, Ciudad Real. SPAIN

Corresponding author. Justo Lobato (justo.lobato@uclm.es)

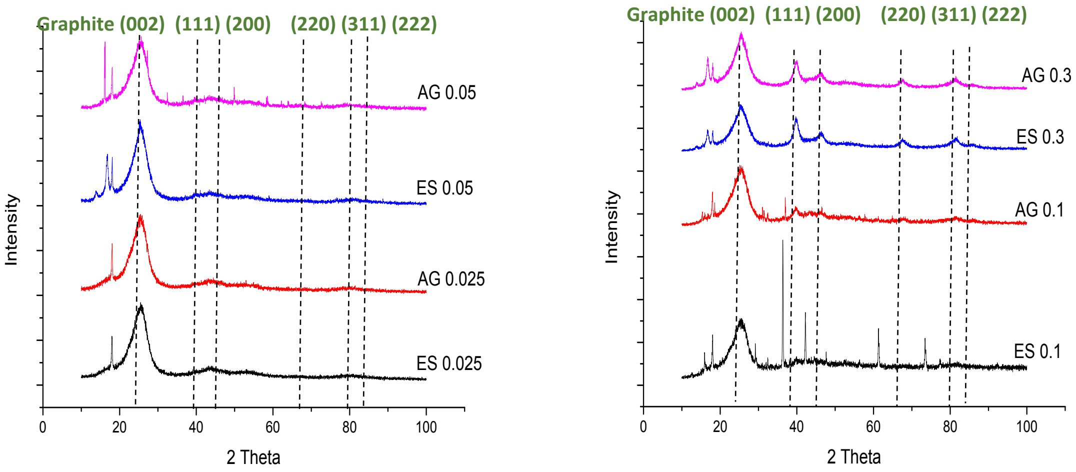

Figure S1: XRD pattern of the GDEs after 500 cycles

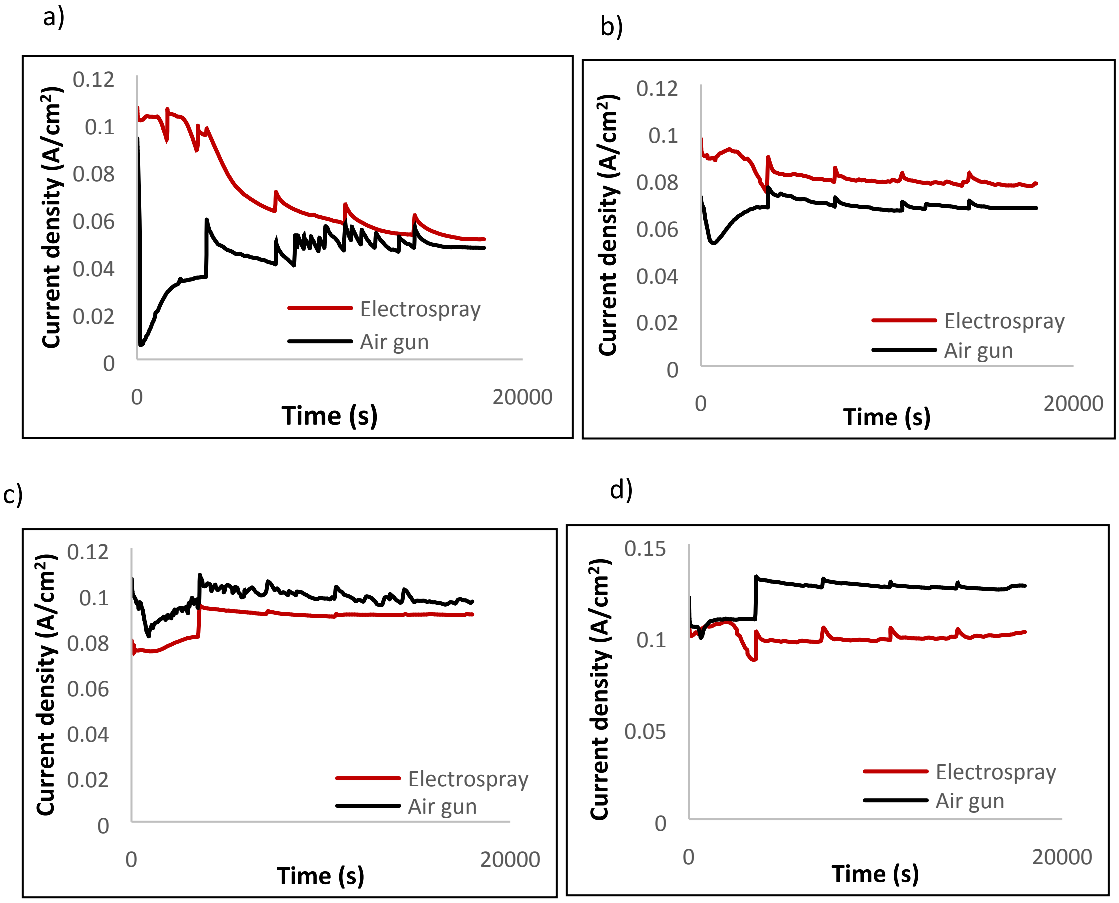

Figure S2: Chronoamperograms of electrodes in working electrolyte for SO_2 oxidation at 1V for different Pt loading (a) 0.025 mg_{Pt}/cm^2 , (b) 0.05 mg_{Pt}/cm^2, (c) 0.1 mg_{Pt}/cm^2 and (d) 0.3 mg_{Pt}/cm^2

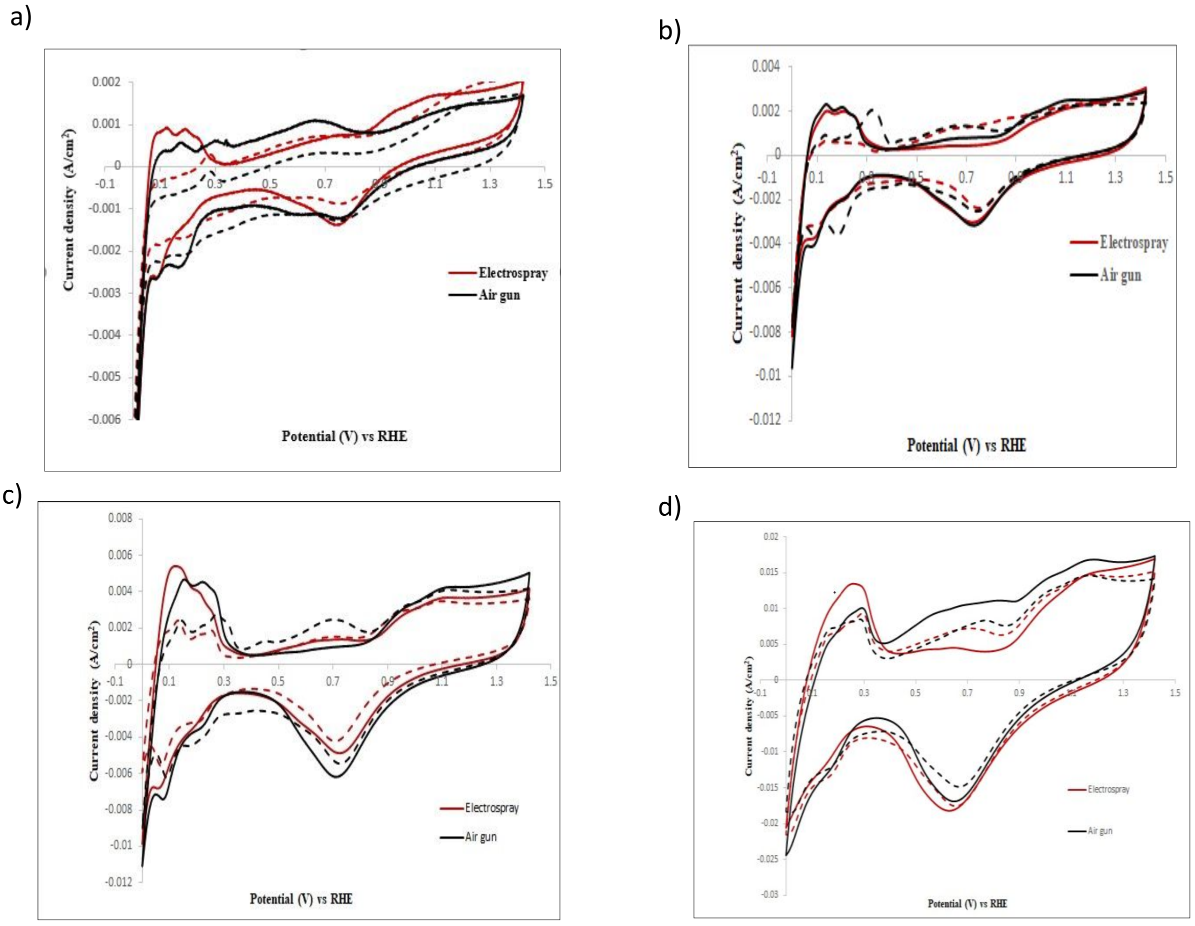

Figure S3: Cyclic voltammerties of electrodes in in nitrogen-saturated 1M H_2SO_4 before (solid line) and after (dashed line) SO_2 oxidation for different Pt loading (a) 0.025 mg_{Pt}/cm^2, (b) 0.05 mg_{Pt}/cm^2, (c) 0.1 mg_{Pt}/cm^2 and (d) 0.3 mg_{Pt}/cm^2

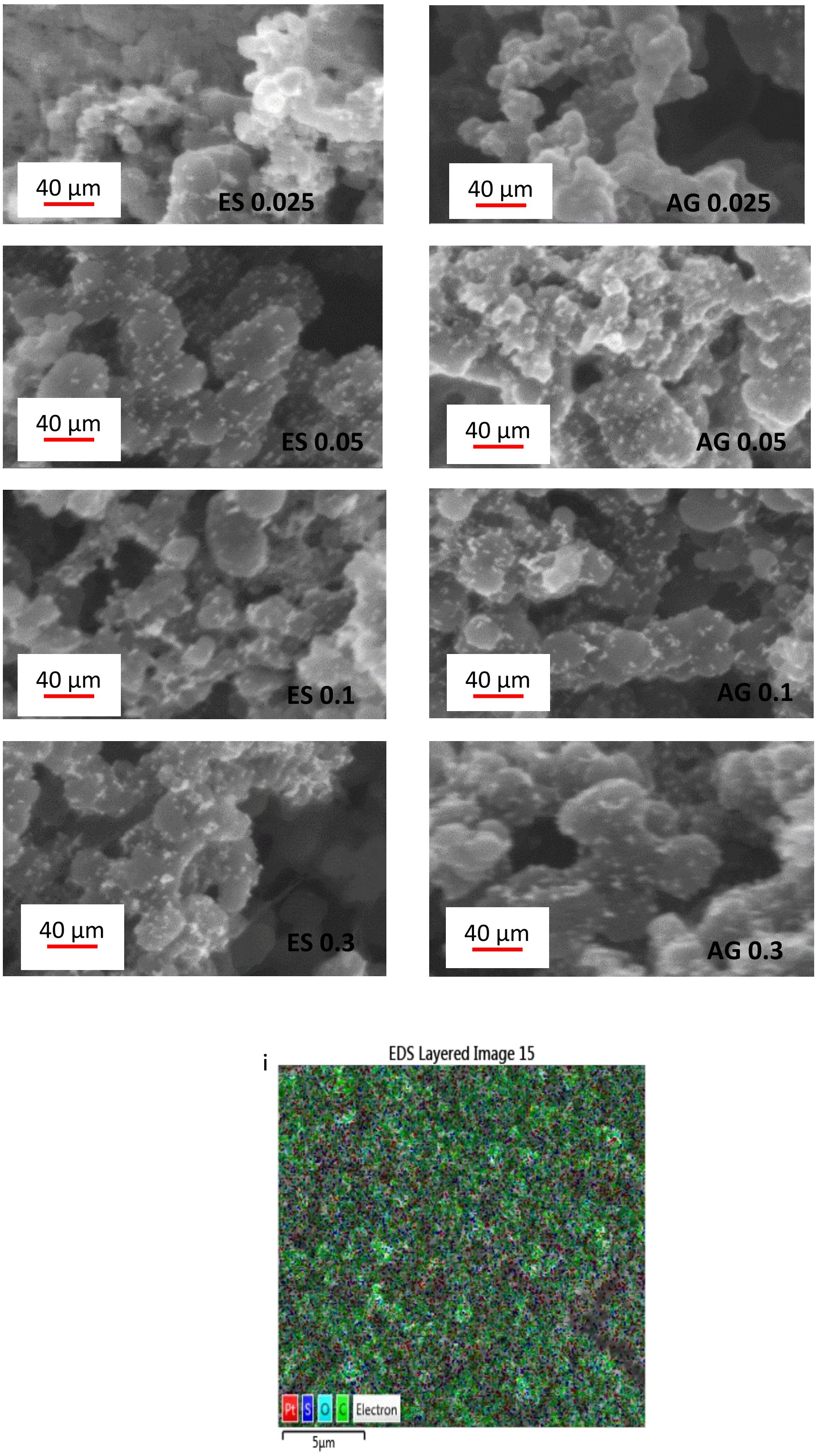

Figure S4: (a-h) SEM images of the catalyst layers prepared with electrospray and air gun with different platinum loading after SO_2 stability test; (i) EDS picture of the surface of catalyst layer after SO_2 oxidation

Table S1: Charge transfer resistance Rct and ohmic resistance Rs for the different GDEs after chronoamperometry tests.

Electrodes	Rct (Ω)	Rs (Ω)
0.025 ES	6.13	6.9
0.025 AG	6.29	7.28
0.05 ES	2.01	9.05
0.05 AG	2.62	9.89
0.1 ES	1.24	8.94
0.1 AG	1.31	7.8
0.3 ES	0.67	7.89
0.3 AG	0.8	6.79
